# Equity impact of minimum unit pricing of alcohol on household health and finances among rich and poor drinkers in South Africa

**DOI:** 10.1136/bmjgh-2021-007824

**Published:** 2022-01-06

**Authors:** Naomi Gibbs, Colin Angus, Simon Dixon, Charles DH Parry, Petra S Meier, Micheal Kofi Boachie, Stéphane Verguet

**Affiliations:** 1 School of Health and Related Research, University of Sheffield, Sheffield, UK; 2 Priority Cost Effective Lessons for Systems Strengethening, South Africa (PRICELESS SA), School of Public Health, Faculty of Health Sciences, University of Witswatersrand, Johannesburg, South Africa; 3 Alcohol Tobacco and Other Drug Use Research Unit, South African Medical Research Council, Cape Town, South Africa; 4 MRC/CSO Social and Public Health Sciences Unit, University of Glasgow, Glasgow, Glasgow, UK; 5 Department of Health Policy Planning and Mangement, School of Public Health, University of Health and Allied Sciences, Ho, Volta Region, Ghana; 6 Department of Global Health and Population, Harvard T.H. Chan School of Public Health, Boston, Massachusetts, USA

**Keywords:** public health, epidemiology, health economics, mathematical modelling, health policy

## Abstract

**Introduction:**

South Africa experiences significant levels of alcohol-related harm. Recent research suggests minimum unit pricing (MUP) for alcohol would be an effective policy, but high levels of income inequality raise concerns about equity impacts. This paper quantifies the equity impact of MUP on household health and finances in rich and poor drinkers in South Africa.

**Methods:**

We draw from extended cost-effectiveness analysis (ECEA) methods and an epidemiological policy appraisal model of MUP for South Africa to simulate the equity impact of a ZAR 10 MUP over a 20-year time horizon. We estimate the impact across wealth quintiles on: (i) alcohol consumption and expenditures; (ii) mortality; (iii) government healthcare cost savings; (iv) reductions in cases of catastrophic health expenditures (CHE) and household savings linked to reduced health-related workplace absence.

**Results:**

We estimate MUP would reduce consumption more among the poorest than the richest drinkers. Expenditure would increase by ZAR 353 000 million (1 US$=13.2 ZAR), the poorest contributing 13% and the richest 28% of the increase, although this remains regressive compared with mean income. Of the 22 600 deaths averted, 56% accrue to the bottom two quintiles; government healthcare cost savings would be substantial (ZAR 3.9 billion). Cases of CHE averted would be 564 700, 46% among the poorest two quintiles. Indirect cost savings amount to ZAR 51.1 billion.

**Conclusions:**

A MUP policy in South Africa has the potential to reduce harm and health inequality. Fiscal policies for population health require structured policy appraisal, accounting for the totality of effects using mathematical models in association with ECEA methodology.

**Key questions**

**What is already known?**

- Alcohol pricing policies, such as taxation and minimum unit pricing (MUP), are consistently recommended by the WHO as one of the most cost-effective measures governments can use to reduce alcohol harm.
- Two recent South African studies have estimated that MUP would be an effective policy in the South African context.
- Pricing policies on harmful products often face criticism for their potentially disproportionate financial burden imposed on the poorest socioeconomic groups.

**What are the new findings?**

- This study estimates that the policy is regressive if analysed using only alcohol consumption expenditures.
- However, we demonstrate that health impacts and other financial outcomes such as avoiding catastrophic health expenditures follow a pro-poor distribution.
- We also demonstrate healthcare cost savings to the government which could potentially be redistributed to further support poorer groups.

**What do the new findings imply?**

- Pricing policies cannot be judged merely by financial regressivity of the consumption expenditures.
- Structured policy appraisal accounting for the totality of effects using mathematical models in association with extended cost-effectiveness analysis methodology can support decision-makers who must make trade-offs across relevant domains.

## Introduction

In 2019, alcohol use was identified as the eighth highest risk factor for mortality in South Africa.^1^ Despite the fact that the prevalence of drinking (and of heavy drinking) increases with wealth, there is an inverse relationship with alcohol harm, with lower socioeconomic groups experiencing the greatest harms.^2^ In South Africa, alcohol harm is wide-reaching, encompassing non-communicable diseases, injuries and infectious diseases. There are high levels of abstinence (82/46% among women/men) coupled with high levels of heavy episodic drinking among those who drink.^3^ As a result of the heavy episodic drinking, the alcohol harm profile contains significant levels of alcohol-related violence and road injury.^1^ South Africa also has a high HIV prevalence (14%^4^) in which alcohol plays a role via increasing risky sexual behaviour and reducing treatment adherence.

Pricing policies are consistently recommended as one of the most cost-effective strategies in reducing the burden of alcohol.^5^ South African research has found that fiscal policies are effective in improving population health including raising excise taxes on beer^6^ and levying a tax on sugar-sweetened beverages.^7 8^ The South African government has used high excise tax increases on tobacco since 1994 which effectively reduced consumption.^9^

Taxation is the most common pricing policy utilised in combating alcohol harm but minimum unit pricing (MUP) is increasing in profile and has been adopted by a number of jurisdictions around the world, including Scotland, Wales, Australia’s Northern Territory and Russia^10 11^ and is now being considered by the Western Cape provincial government in South Africa.^12^ MUP is a policy whereby a retail floor price is set contingent on the alcohol content of the drink. This means the policy targets the very cheapest alcohol on the market, consumed primarily by the heaviest and often the poorest, drinkers. This is in contrast to the effect of raising excise taxes which increases prices across the price distribution in a more uniform manner.

The current South African alcohol taxation system is inconsistent, with wine and traditional African beer taxed per litre of drink (ZAR4.4/ZAR0.8 for wine/African beer) and malt beer and spirits taxed per litre of absolute alcohol (ZAR106.6/ZAR213.1 for beer/spirits).^13^ This taxation system results in wine and traditional beer benefiting from much lower rates of tax by volume of absolute alcohol. There are currently no minimum prices in effect. Two recent policy appraisal studies have estimated that MUP would be an effective policy in the South African context to reduce overall consumption and harm, particularly among the heaviest drinkers.^14 15^

South Africa experiences high levels of income inequality and around 45% of households were in receipt of at least one form of social grant in 2015.^16^ In addition, income-related health inequality has increased as a result of COVID-19.^17^ Against this backdrop, a significant equity concern relating to pricing policies such as MUP for South Africa, and many other countries, is their potentially financially regressive nature. That is, the ratio of increase in alcohol expenditures to income would become smaller as wealth or income increases, and as such poor income groups could bear a disproportionate financial burden following MUP implementation.^18 19^ However, this partial view fails to account for the broader set of financial consequences following enforcement of pricing policies including MUP. Importantly, these financial consequences include, for example, the reductions in out-of-pocket (OOP) costs associated with decreased alcohol-related disease treatment costs and the potentially ensuing medical impoverishment for drinkers and their families, as well as household income savings associated with reduced absenteeism tied to alcohol-related disease. A wider perspective would also consider non-financial flows (eg, health benefits associated with reduction in alcohol-related disease morbidity and mortality) where low-income groups are likely to benefit more due to their disproportionate disease burden at baseline. Finally, any increase in revenue to the government, either through taxation or through savings to the healthcare sector budget, are likely to result in a progressive redistribution of resources, such that the increased budget is used to make payments or provide services which benefit the lowest income groups.^20 21^

In summary, examining a broad range of effects, along both the health and financial dimensions, of pricing policies for harmful products (eg, alcohol, tobacco and sugary drinks), is absolutely essential to enable the comprehensive assessment of their equity and redistributive impact. The model used in this study was based on stakeholder engagement which shaped the choice of policy, outcomes and subgroups. This is essential to addressing a contextually defined concern for equity. ECEA provides a helpful framework for exploring equity but does not replace stakeholder engagement which may highlight the need for additional and complementary analyses that measure equity impacts in different ways for example including a broader range of outcome measures or alternative subgroups of interest.

In this paper, we build on a recently published modelling study of MUP in South Africa,^14^ which details an epidemiological policy appraisal model. We draw from extended cost-effectiveness analysis (ECEA) methods,^22–24^ which enable the equity impact evaluation of health policies along socioeconomic groups, so to exhibit a broad range of outcomes and the potential equity impact of MUP for alcohol in South Africa.

## Methods

### General approach

**Figure 1:**
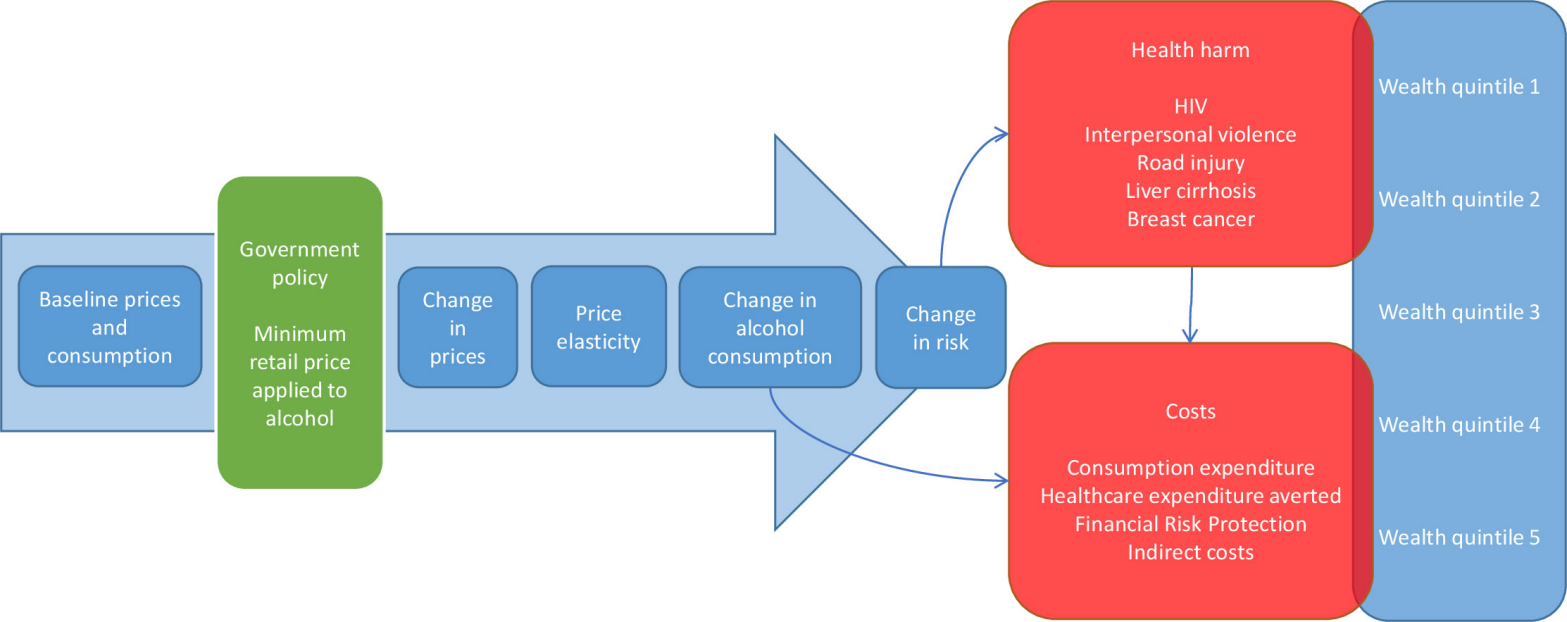
Description of the Minimum Unit Pricing model contextualised to South Africa and expanded via the extended cost-effectiveness analysis framework. Adapted from: Gibbs *et al.*^14^ Licensed under Creative Commons Attribution (CC BY 4.0) available at: https://creativecommons.org/licenses/by/4.0/

We build on a recent MUP model contextualised to South Africa that is described in great detail elsewhere.^14^ The model uses a public health epidemiological model that can be best described as a comparative risk assessment model using multistate life tables, over a 20-year time horizon.^25^ We expand this MUP model in applying the ECEA framework (figure 1), specifically this requires the addition of a number of new variables (OOP costs, mean wages, cases of catastrophic health expenditures (CHE) averted) and increased disaggregation of inputs by wealth quintile beyond that used in the original model (eg, the incorporation of healthcare utilisation rates). ECEA examines the impact of policy along both health and financial dimensions:^24^ (i) health gains, in other words the number of deaths related to a selection of alcohol-related diseases averted; (ii) financial gains, which include the amount of OOP costs tied to treatment of alcohol-related diseases averted and their associated financial risk protection (FRP) benefits (eg, corresponding to the prevention of medical impoverishment from OOP treatment costs of alcohol-related diseases). All health and financial dimensions are then displayed in a disaggregated manner across socioeconomic groups (eg, wealth quintiles) so as to point to the potential equity impact of the policy. We populate our expanded model while drawing from multiple sources of data disaggregated across South African socioeconomic groups including household surveys, administrative data sets and the published literature (table 1; online supplemental appendix sections 1–2).

10.1136/bmjgh-2021-007824.supp1 Supplementary data

**Table 1:** Data inputs and corresponding sources used in modelling of the equity impact of the minimum unit pricing policy for alcohol in South Africa

Input	Wealth quintiles (QI=poorest)*					Source
	QI	QII	QIII	QIV	QV	
Alcohol consumption, prices and elasticities						
Prevalence of drinking	27%	30%	33%	35%	38%	SA DHS 2016
Prevalence of heavy drinking (more than 15 standard drinks per week)	14%	14%	16%	17%	20%	SA DHS 2016
Mean individual baseline consumption (standard drinks per week)	20.6	21.4	20.9	21.7	20.7	SA DHS 2016 calibrated to Euromonitor
Mean price per standard drink						International Alcohol Control Study (2014) adjusted for inflation to 2018 prices Gibbs *et al*^14^
Moderate	R9.1	R9.1	R9.1	R11.6	R11.6	
Occasional binge	R8.0	R10.0	R10.1	R13.4	R11.1	
Heavy	R7.8	R9.7	R9.2	R10.6	R12.8	
Price elasticity by drinker groups†						Van Walbeek and Chelwa^41^ authors’ calculations (webappendix section 3) Gibbs *et al*^14^
Moderate	−0.53	−0.53	−0.31	−0.31	−0.31	
Occasional binge	−0.29	−0.29	−0.17	−0.17	−0.17	
Heavy drinkers	−0.24	−0.24	−0.14	−0.14	−0.14	
Share of disease at baseline‡						
HIV	20%	36%	32%	9%	3%	Authors’ calculations using GHS 2018
Intentional injury road injury Liver cirrhosis	9%	29%	26%	26%	10%	Authors’ calculations using GHS 2018
Breast cancer	7%	7%	22%	18%	47%	Authors’ calculations’ using GHS 2018
Disease-related expenditure and utilisation						
Proportion of disease-related expenditures paid as OOP	21%	18%	41%	56%	82%	Saxena et al.^22^
HIV utilisation rates	63%	71%	69%	60%	89%	Authors’ calculations using GHS 2019 (webappendix section 5)
Trauma care utilisation rates—intentional injury	39%	40%	40%	40%	47%	Authors’ calculations using GHS 2019 data plus Matzopoulos et al.^42^ (webappendix section 5)
Trauma care utilisation rates—road injury	18%	19%	18%	18%	22%	Authors’ calculations using GHS 2019 data; Matzopoulos et al.^42^ (webappendix section 5)
Healthcare utilisation rates—liver cirrhosis	52%	55%	54%	53%	63%	Authors’ calculations using GHS 2019 (webappendix section 5)
Healthcare utilisation rates—breast cancer	52%	56%	50%	68%	89%	Authors’ calculations using GHS 2019 (webappendix section 5)
Labour and productivity						
Labour force participation	62%	50%	55%	64%	74%	Authors’ calculations using GHS 2019 data
Annual income per capita (ZAR)	6100	27 400	49 300	95 600	408 900	Authors’ calculations using GHS 2019 data deflated to 2018
Absenteeism (days per year)						
HIV	14	14	14	14	14	Maffessanti and Lee-Angell^43^
Intentional injury	10	10	10	10	10	Bola *et al*^44^
Road injury	18	18	18	18	18	Parkinson *et al*^45^
Liver cirrhosis	6	3	3	3	3	Matzopoulos *et al*^46^
Breast cancer	6	6	6	6	6	Tangka *et al*^47^ (webappendix section 6)

*Wealth quintiles defined as the asset index measure provided in the SA DHS data; authors used an ordered choice regression model to predict wealth quintiles for the International Alcohol Control (IAC) data set; income quintiles used as a proxy for wealth quintiles in GHS data.

†Drinker groups: moderate=less than 15 standard drinks per week; occasional binge=less than 15 drinks per week but drinks more than five on at least one occasion; heavy=15 or more standard drinks per week. Standard drink=12 g or 15 mL of pure ethanol.

‡Share of disease at baseline indicates how the cases of the disease/injury conditions are distributed among the quintiles.

DHS, Demographic and Health Survey; GHS, General Household Survey; OOP, out-of-pocket; SA, South Africa.

Importantly, we examine a broad range of effects of a MUP policy for alcohol, along both the health and financial dimensions and across socioeconomic groups, in South Africa. We track the following outcomes, as a result of MUP, across national wealth quintiles: the impact on alcohol consumption; the change in mortality attributed to alcohol-related diseases (five major alcohol-induced conditions are included: HIV, intentional injury, road injury, liver cirrhosis and breast cancer); the change in alcohol consumption expenditures; the reduction in expenditures, both for the government and households (ie, OOP cost savings), associated with treatment of alcohol-related diseases, and the FRP benefits for households linked to reductions in those OOP costs for treatment of alcohol-related diseases and the household savings tied to indirect costs (associated with absenteeism) following the decreased burden of alcohol-related diseases.

### Policy simulation

A MUP policy is where the government legislates for a retail floor price based on the alcohol content of the drink, in this case ZAR 10 (US$0.76) for one standard drink (12 g of pure alcohol, ie, 330 mL beer or a 125 mL glass of wine), a level chosen by policymakers. It pushes all prices currently below that level up to that level. We assume all prices above that level remain unchanged. This results in a price increase experienced by the consumer (dependent on how much cheap alcohol they purchase) which, dependent on their price responsiveness (measured by their price elasticity of demand) will change their purchasing decisions. All these simulations are disaggregated across South African wealth quintiles.

### Modelling features

#### Price, consumption and health impact

To model the relationship between alcohol price and consumption, we first estimate the preintervention mean and peak alcohol consumption at the individual level. The base year for the model is 2018 and all monetary inputs are indexed to this year. The model includes the adult population only (those aged 15 years and older) with each individual classified as an abstainer or drinker. Drinkers are then classified as moderate (consumption of <15 standard drinks per week), occasional binge (consumption of <15 drinks per week but drinks>5 drinks on one occasion) and heavy (≥15 drinks per week). The change in price from the policy is translated into a change in individual consumption using an elasticity of demand for alcohol which varies by drinker type and wealth group (online supplemental appendix sections 3–4). Adjustments are made for individuals increasing consumption of homebrew (about 4% of all reported alcohol consumption in the survey was homebrew). Individual-level changes in consumption and spending are then aggregated to get results at the wealth quintile level at baseline and under MUP. Increases in individual consumption expenditures are projected forward and discounted at 5% per year, a rate recommended by South Africa’s Department of Health^26^ before being aggregated across quintiles.

Given that depending on the health condition, there can be a delay between changes in alcohol consumption and changes in health risks, the model uses a 20-year time horizon to assess the full impact of MUP on disease or injury outcomes. Our model calculates relative risks (RR) for each of five major conditions that can be associated with alcohol consumption: HIV, intentional injury, road injury, liver cirrhosis and breast cancer. It uses individual alcohol consumption at baseline and at ZAR 10 MUP. The five conditions were chosen by stakeholders during the original model development process.^14^ Potential impact fractions (PIFs) were calculated by dividing RR under MUP by RR at baseline. Using these PIFs and projecting the population forward 20 years, we could compute the number of deaths averted by MUP. These projected populations (no MUP vs ZAR 10 MUP) were then combined with the probability of having the condition (disease or injury) to estimate disease-specific cases and deaths.^14^

### Healthcare expenditures, OOP costs and financial risk protection

The prevalence of each condition (disease or injury) under each policy scenario was multiplied by the proportion who would then go on to receive treatment using condition-specific and quintile-specific healthcare utilisation rates (table 1). Condition-related treatment unit cost estimates were sourced from the literature and adjusted for inflation^27^ (where necessary) to reach the baseline year of 2018. All future costs were discounted at 5% per year.^26^ The multiplication of those condition-related treatment unit costs by the corresponding condition-related utilisation rates would yield expected treatment costs for each condition.

Healthcare in South Africa is delivered via a mix of public (with contributions from the patients determined on a sliding pay scale) and private providers and health insurance mechanisms. As such, the reduction in the burden of alcohol-related conditions/diseases will lead to decreases in healthcare costs for both the South African government (‘government savings’) and households (‘OOP cost savings’). The partition of these healthcare cost savings into either government savings or OOP cost savings was attributed by using the mean shares (percentages) for each wealth quintile using previously published estimates.^22 28^

Subsequently, FRP benefits associated with household cost savings were derived for each quintile. The measure of FRP used was the number of cases of CHE averted by MUP. A case of CHE would be counted when, for an instance of alcohol-related condition seeking care, the disease-related OOP treatment costs averted would exceed 10% of total annual household income.

Finally, we computed indirect costs using the human capital approach. This included an estimation of the value of lost (productive) time, using gross wage as the measure of value, as a result of the morbidity associated with the five conditions enumerated above. Indirect costs were calculated by applying the number of lost days due to disease/injury per year by the mean daily wage by income quintile, taking into account the labour force participation by quintile and prevalence of disease. The evidence relating productivity and alcohol remains inconclusive and so was not modelled.^29^

### Sensitivity analyses

We conducted multiple univariate sensitivity analyses on key parameters including: price elasticities; CHE thresholds and wage rates. For price elasticities, we explored two alternative scenarios. First, we removed the wealth gradient from the price elasticity estimates using −0.40 to –0.22, and −0.18 for moderate, occasional binge and heavy drinkers, respectively. Second, we used alternative price elasticities estimated by Van Walbeek and Blecher^30^ using National Income Dynamic Study data for two subsets of the population, the top and bottom 50% of households by total expenditures. We applied −0.86 to quintiles I and II and −0.50 for quintiles III, IV and V (to be conservative). These estimates are closer to other South African alcohol elasticity estimates including −0.80 and −0.75.^30^ For the estimation of CHE cases, we used alternative thresholds of 25% and 40% of income. Finally, we applied the South African minimum wage (ZAR20.8) per hour across all quintiles to calculate productivity losses. This avoided applying less value to those on lower wages, in the calculation of indirect costs.

### Display of findings

All results are given in ZAR (R). Headline results quoted in the text are also converted into US$ using the exchange rate at 2018 of R13.2 per US$.^31^ All computations were realised using R statistical software (code available here). Our results are disaggregated by wealth quintile for the following outcomes: deaths averted attributed to alcohol-related diseases and injuries; net change in alcohol expenditures; government cost savings; household OOP cost savings and number of CHE cases averted; and indirect cost savings.

## Results

**Figure 2:**
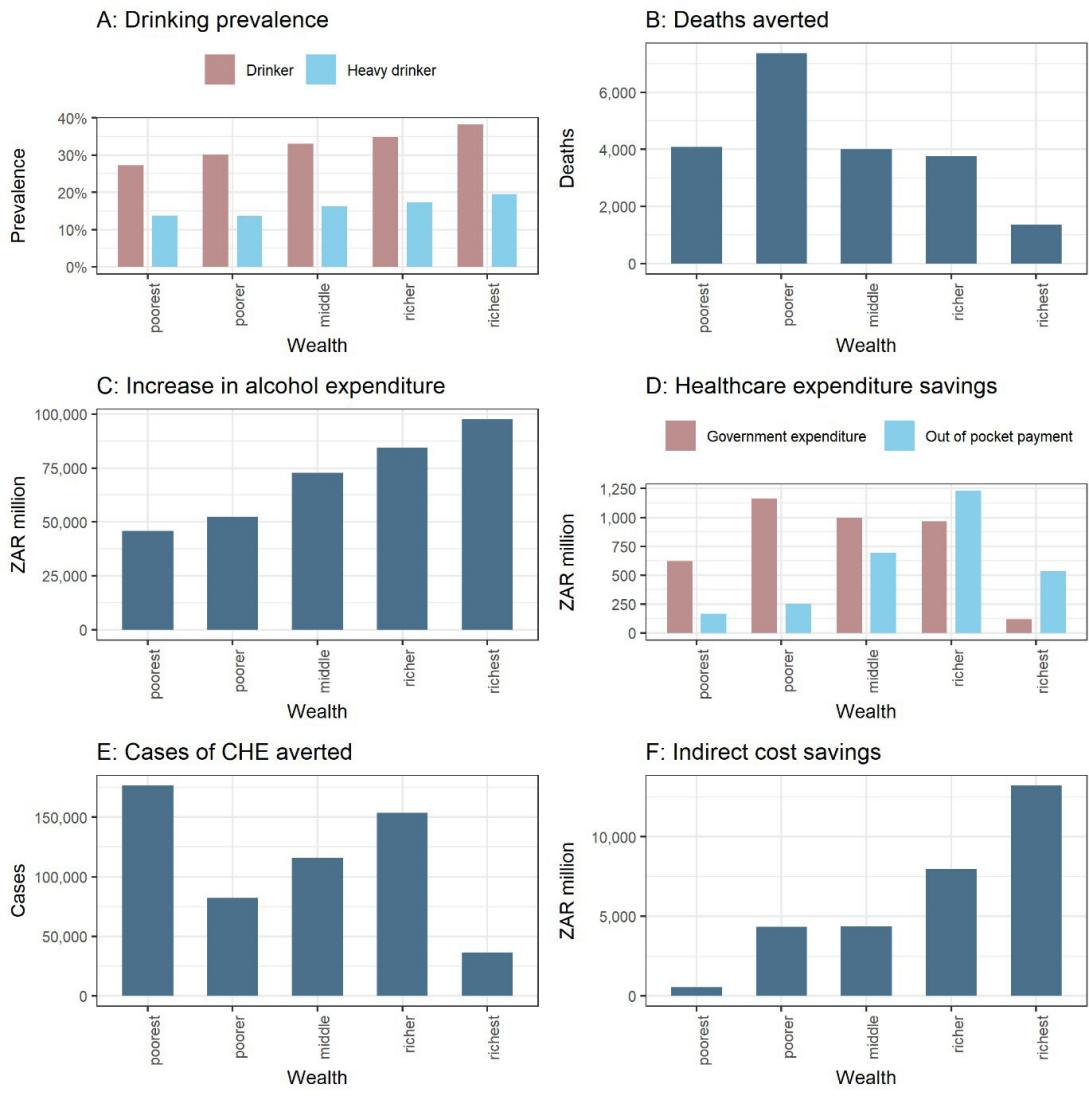
Estimated distributions, across wealth quintiles, of the health and financial outcomes following implementation of Minimum Unit Pricing (MUP) in South Africa. (A), drinking prevalence; panels (B–F) demonstrate the distributional (equity) impact of the policy, all estimates are for a 20-year time horizon; (B), deaths averted; (C), net change in alcohol expenditures; (D), healthcare cost savings (government vs OOP cost savings); (E), cases of catastrophic health expenditures (CHE) averted; (F), indirect costs savings.

First, the reduction in consumption would be substantially more among the poorest than the richest (−7.8% relative decrease vs −3.2%) out of an overall change in consumption of −4.4% (for a R10 MUP). Total deaths averted were estimated at 22 600: the greatest number of deaths averted would accrue to quintile II while overall those benefits would largely be pro-poor with 56% of deaths averted accruing to the bottom two quintiles (table 2; figure 2). This in fact reflects the underlying gradients of the five conditions examined. The smallest effect is for the richest quintile which would accrue only 7% of the total deaths averted.

**Table 2:** Net change in health and financial outcomes across socioeconomic groups for a ZAR10 minimum unit pricing policy in South Africa

	Overall	QI	QII	QIII	QIV	QV
Deaths averted	22 600	4100	7400	4000	3800	1400
Net change in alcohol expenditures (ZAR million)	R353 000	R46 000	R52 000	R72 800	R84 500	R97 600
OOP healthcare cost savings (ZAR million)	R2900	R200	R300	R700	R1200	R500
Government healthcare cost savings (ZAR million)	R3900	R600	R1200	R1000	R1000	R100
Cases of CHE averted	564 700	176 700	82 000	115 900	153 800	36 400
Annual indirect cost savings (ZAR million)	R51 100	R4700	R11 600	R8400	R11 800	R14 700

All results projected over a 20-year time horizon.

Deaths averted and CHE cases averted rounded to the nearest hundred.

Financial outcomes rounded to the nearest hundred million.

CHE, Catastrophic health expenditures; OOP, out-of-pocket; QI, poorest wealth quintile; QV, richest wealth quintile; ZAR/R, South African Rand.

Given the baseline price elasticities of demand for alcohol are relatively inelastic (−0.14 to −0.53), when prices rise, demand would reduce by less in proportionate terms, which leads to increased alcohol expenditures. We estimated increased expenditures of ZAR 353 000 million (US$26 700 million). The poorest would contribute the lowest proportion (about 13%), while the richest the largest (around 28%) of the expenditures (figure 2). Despite the richer quintiles experiencing the smallest percentage increase in alcohol prices (driven by their higher baseline mean price), they would still pay the largest share of increased alcohol expenditures due to their lower price elasticity and higher prevalence of drinking. The policy would be regressive (in the narrow consumption expenditure sense) with the ratio between increased expenditures on alcohol and income estimated to be 27.0, 5.9, 3.9, 2.2 and 0.5% from the poorest to the richest quintile.

In addition, we estimated a reduction in OOP healthcare costs of about ZAR 2.9 billion (US$0.22 billion) and government cost savings of approximately ZAR 3.9 billion (US$0.30 billion). The relative distribution of these costs across quintiles reflects the sliding scale of payments charged for healthcare in South Africa with the bottom two quintiles paying the least amount of OOP costs (21% and 18% shares, respectively), consequently they would see the smallest OOP savings (figure 2).

Furthermore, we found that 564 700 CHE cases would be averted. Quintile I would accrue the highest number of CHE cases due to their very low incomes meaning even small OOP treatment costs would lead to CHE cases. Quintile IV also realises high numbers of CHE cases averted as the rise in income is offset by the reduction in government subsidy for healthcare costs incurred. As expected, quintile V would accrue the smallest number of CHE cases averted, with only about 6% of all cases (figure 2).

Finally, the savings in indirect costs were estimated at ZAR 51 100 million (US$3900 million). There is generally a positive gradient across the quintiles driven by both the increasing labour participation and increasing wage rate (figure 2).

### Sensitivity analyses

**Figure 3:**
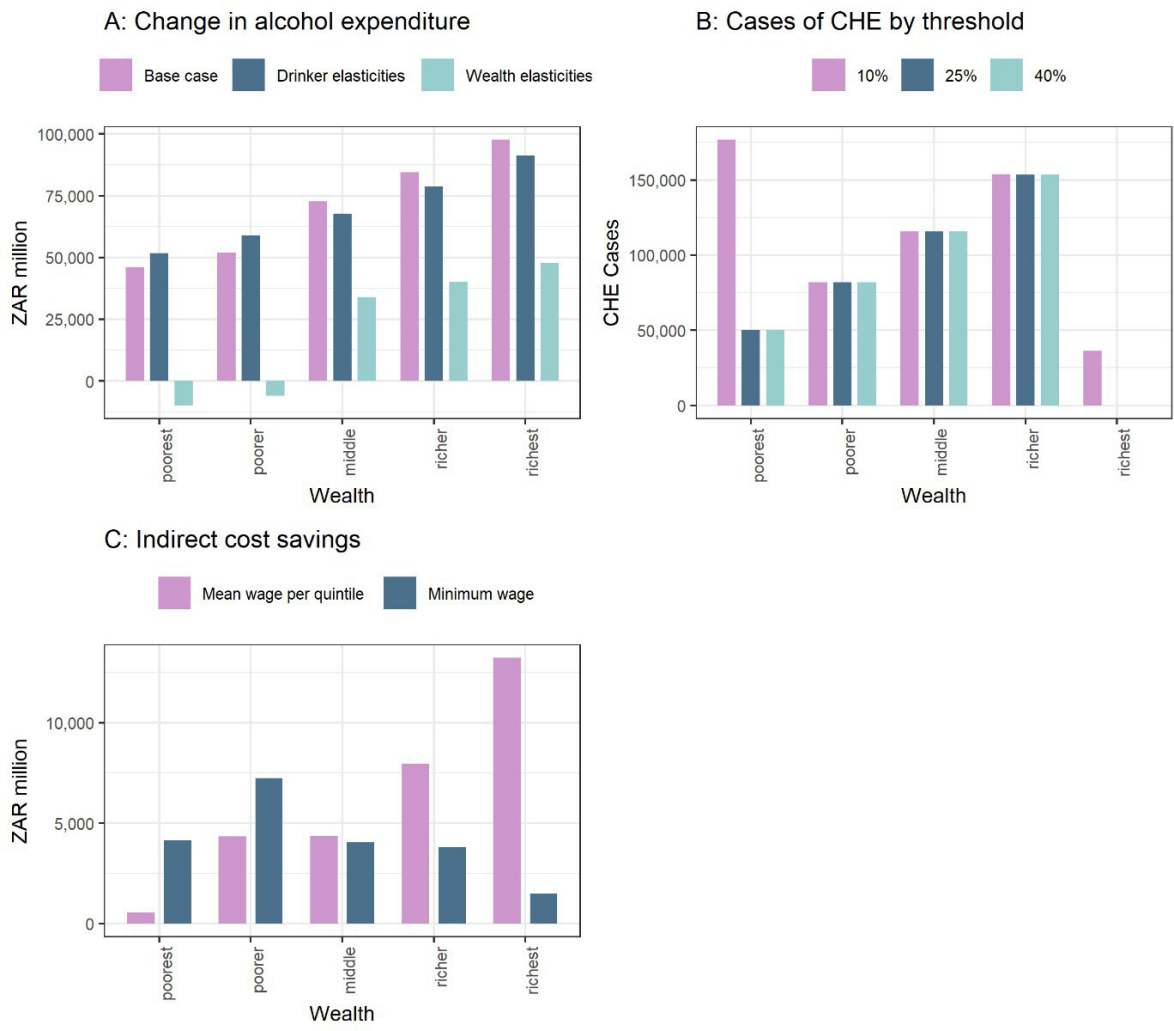
Distributional (equity) impact of the sensitivity analyses. All estimates are for a 20-year time horizon. A, change in alcohol expenditures comparing three different price elasticity sets; B, cases of catastrophic health expenditures (CHE) using alternative thresholds; C, indirect costs savings.

A key driver for the results is the price elasticities. We explored two alternative scenarios. First, using −0.40 (moderate), −0.22 (occasional binge) and −0.18 (heavy drinkers), without applying any wealth gradient, the resulting consumption impact would be reduced but remain pro-poor (−5.7% for the poorest vs −4.1% for the richest). Second, using −0.86 for quintiles I and II and −0.50 for quintiles III to V would result in a reduction in alcohol expenditures, compared with baseline, for quintiles I and II (table 3; figure 3).

**Table 3:** Key results for the sensitivity analyses (over a 20-year time horizon)

Sensitivity analysis: elasticities, CHE thresholds, wage rates	Overall	QI	QII	QIII	QIV	QV
Panel A: varying elasticities						
Drinker groups adjusted for wealth (base case)						
Deaths averted	22 600	4100	7400	4000	3800	1400
Change in consumption expenditures for drinkers (ZAR million)	R353 000	R46 000	R52 000	R72 800	R84 500	R97 600
No wealth gradient: −0.4/–0.22/−0.18 moderate/occasional binge/heavy drinkers						
Deaths averted	18 717	1500	6500	4400	4500	1800
Change in consumption expenditures for drinkers (ZAR million)	R348 600	R51 800	R58 900	R67 800	R78 800	R91 200
No drinker gradient: –0.86/–0.5 poorest–poorer/middle–richest						
Deaths averted	52 400	11 800	18 400	10 600	8300	3400
Change in consumption expenditures for drinkers (ZAR million)	R106 000	–R9900	–R5900	R33 900	R40 200	R47 800
Panel B: cases of CHE averted at 10%, 25% and 40% thresholds						
10% (base case)	564 700	176 700	82 000	115 900	153 800	36 400
25%	401 300	50 200	81 900	115 700	153 600	0
40%	401 300	50 200	81 900	115 700	153 600	0
Panel C: indirect cost savings (ZAR million) for baseline and minimum wage						
Indirect costs savings using mean wage by quintile (base case)	R51 100	R4700	R11 600	R8400	R11 800	R14 700
Indirect cost savings using minimum wage applied across all quintiles	R20 700	R4100	R7200	R4100	R3800	R1500

Deaths averted and CHE cases averted rounded to the nearest hundred.

Financial outcomes rounded to the nearest hundred million.

A, change in deaths averted and alcohol consumption expenditures for three distinct price elasticity sets; B, cases of catastrophic health expenditures (CHE) with 10/25/40% thresholds; C, indirect cost savings using wage by quintile versus minimum wage across the quintiles.

QI, poorest wealth quintile; QV, richest wealth quintile; ZAR/R, South African Rand.

When the CHE threshold was varied from 10% to either 25% or 40%, the number of CHE cases averted would fall to 401 300 for both alternative thresholds (from 564 700 previously) (table 3). This is driven primarily by a change to the number of CHE cases averted in quintile I (figure 3).

Finally, we estimated indirect cost savings using the minimum wage (ZAR 20.8) across all quintiles instead of the mean wage per quintile in the base case (table 3). As expected, the total indirect cost savings would decrease and the benefits shift towards the poorer quintiles (figure 3).

## Discussion

We demonstrated in this paper that a ZAR 10 MUP policy could significantly reduce alcohol consumption in South Africa, with far greater reductions for the poorest than the richest wealth quintiles. Importantly, we also determined that the number of alcohol-related deaths averted would largely be pro-poor, with 56% of the total deaths averted accruing to the bottom two quintiles. The increase in alcohol expenditures would increase with wealth. However, when calculated as a proportion of income, the increase in alcohol expenditures is greatest for the poorest, which was to be expected given the large income inequalities in South Africa.

Additionally, reductions in alcohol-related disease healthcare expenditures (approximately ZAR 6.8 billion or US$0.52 billion) would be very substantial with consequent government cost savings and household OOP cost savings reflecting South Africa’s health system financing structure.^32^ Importantly, FRP benefits would be large with CHE cases averted concentrated between quintiles I and IV. Indirect cost savings of ZAR 51 100 million (US$3900 million) would be distributed towards the rich due to their higher labour market participation rates coupled with higher wage rates.

Despite this range of positive impacts, the increases in alcohol expenditures relating to MUP are regressive in the sense that the increase in alcohol expenditures relative to income is 27% for the lowest income quintile, compared with 0.5% in the richest quintile. The basic reason for this is that the currently available estimates of price elasticity show the demand for alcohol to be inelastic; that is, consumption reductions following a price change are small, thereby increasing expenditures. When increased expenditures are coupled with a very unequal distribution of income, then the resulting expenditures become regressive. If the elasticity estimates are correct, this regressive component of MUP is not going to change. However, our modelling provides wider information beyond this natural consequence of a basic economic principle. Importantly, it quantifies the trade-offs that faces the South African government when considering MUP. As we show, MUP is expected to have many benefits, both in absolute terms and in equity terms, and our results provide the information needed to assess whether the overall effects are considered socially desirable (or not). Although the policy might be regressive in a narrow economic sense (yet, this is less clear if you consider CHE), it is almost certainly progressive in a wider health context. In addition, the formulation of a subset of these findings in the form of an ECEA provides a simpler way to communicate this information to decision-makers. Also, but beyond the scope of this paper, by knowing the scale and nature of all these impacts it is possible to use our model to design auxiliary policies that will mitigate the regressivity in relation to alcohol expenditures, for example, redirecting the increased tax revenues and healthcare budget savings associated with MUP to lower socioeconomic groups.

It is also important to consider these findings in the context of South Africa’s high abstinence rates. In every quintile, self-reported abstainers are in the vast majority, particularly among women (82%). Non-drinkers will experience benefits from a reduction in others’ drinking via reductions in intimate partner violence, fetal alcohol syndrome and other forms of crime and violence,^33 34^ as well as reductions in household OOP treatments (which we document in this paper). There may also be benefits from a reduction in alcohol initiation. However, non-drinkers may also suffer as a result of the policy through the impact on the household budget with resources being diverted to pay for alcohol (ie, crowding-out). This concern is common across pricing policies of unhealthy goods and further reinforces the importance of the pro-poor use of any generated tax revenues or healthcare cost savings.^35^ The introduction of a MUP policy would benefit from a comprehensive monitoring and evaluation programme including qualitative interviews with households comprising of at least one heavy drinker to assess this impact and possibly also tracking the impact of conditions shown during the COVID-19 pandemic to particularly affect the healthcare system, such as alcohol-related trauma admissions in South Africa.^36^

Our sensitivity analyses employing alternative elasticities highlight the importance of these critical input parameters on the distributional impact of MUP. If the poorer quintiles are highly price elastic (as in the scenario with −0.86), then the model estimates cost savings for these groups. This would mean MUP would cease to be regressive in terms of consumption expenditures. This aligns broadly with international evidence (from both modelling studies and empirical evaluation) which suggests limited regressive effects, or in some cases financial gains from reduced consumption expenditure, for the poorest groups.^11 37 38^ We recommend further research to estimate elasticities for poorer drinkers, disaggregated by drinker type group.

In addition, alternative alcohol pricing policies such as moving to a consistent volumetric tax system (in which all alcohol is taxed based on litres of absolute alcohol) could produce similar results by ‘eliminating’ the cheapest alcohol. In addition, they would provide an increase to the fiscal budget rather than to economic operators. This could theoretically be reinvested in policies such as providing alcohol treatment services to low-income groups. In the case of MUP, any increase in revenue is kept by the retailer which may be seen as supporting business by advocates of the policy, however, the government will also realise some of the benefits via increased taxes.

### Limitations

This research is limited by a number of factors. First, there are inherent limitations associated with the pricing data we used (eg, alcohol being considered as one sole commodity).^14^ Second, our modelling only included five of over 30 wholly or partially alcohol-attributable conditions, and, as such, would only represent a limited proportion of all potential health outcomes and associated healthcare cost savings.^39^ Moreover, we have conservatively estimated healthcare costs: for example, HIV-related costs were estimated only for first line antiretroviral therapy, and including higher HIV costs would likely lead to greater savings in quintiles I and II (with higher HIV prevalence). Third, we were unable to include all costs associated with the diseases and injuries examined, such as transport costs, traditional medicine costs and caregiver costs which may be significant and therefore underestimate the potential cost savings of the policy.^40^ Fourth, we used wealth quintiles based on an asset score of ownership of certain goods and access to facilities such as water and sanitation, while a number of our input parameters (eg, utilisation rates, wages) used income to categorise people into quintiles: this may introduce some small variations although they should broadly correspond.

## Conclusion

This study has demonstrated a complex set of impacts with wealth gradients varying dramatically across the policy relevant health and financial outcome measures. This highlights the critical relevance for structured policy appraisals accounting for the comprehensive impacts of fiscal policies like ‘sin’ or health taxes and pricing policies, which goes beyond the mere assessment of regressivity or progressivity solely based on a narrow income-share accounting definition of price or tax burden.^21^ The ZAR10 MUP policy would be financially regressive in terms of increased alcohol expenditures (despite the richest paying the largest share of the increased expenditures), however, the poorest groups would gain more health benefits (greater numbers of deaths averted) and face an increased chance of avoiding CHE and medical impoverishment. Policymakers must balance a broad range of aggregate and distributional effects along with accompanying trade-offs in order to make socially optimal policy decisions, promote health equity and reduce inequalities.

## Data Availability

Data may be obtained from a third party and are not publicly available. All data sources used in the model are listed in the web appendix. Data may be obtained from a thrid party and are not publically available

## References

[1] (IHME). GBD compare Seattle. WA: IHME, University of Washington, 2019. http://vizhub.healthdata.org/gbd-compare

[2] Probst C, Parry CDH, Wittchen H-U, et al. The socioeconomic profile of alcohol-attributable mortality in South Africa: a modelling study. BMC Med 2018;16:97. 10.1186/s12916-018-1080-0 29936909 PMC6016129

[3] National Department of Health (NHoH) SSAS, South African Medical Research Council (SAMRC), ICF. South African demographic and health survey 2016. Pretoria South Africa: NHoH, Stats SA, SAMRC, and ICF, 2019.

[4] Rebublic of South Africa. Mid-year population estimates, 2019. In: Statistics. South Africa, 2019.

[5] World Health Organization. Global status report on alcohol and health. Geneva: WHO, 2018. https://www.who.int/substance_abuse/publications/global_alcohol_report/en/

[6] Stacey N, Summan A, Tugendhaft A, et al. Simulating the impact of excise taxation for disease prevention in low-income and middle-income countries: an application to South Africa. BMJ Glob Health 2018;3:e000568. 10.1136/bmjgh-2017-000568 PMC5838397 29515917

[7] Manyema M, Veerman LJ, Tugendhaft A, et al. Modelling the potential impact of a sugar-sweetened beverage tax on stroke mortality, costs and health-adjusted life years in South Africa. BMC Public Health 2016;16:405. 10.1186/s12889-016-3085-y 27240422 PMC4886444

[8] Blecher E. Taxes on tobacco, alcohol and sugar sweetened beverages: linkages and lessons learned. Soc Sci Med 2015;136-137:175–9. 10.1016/j.socscimed.2015.05.022 26005761

[9] Linegar DJ, van Walbeek C. The effect of excise tax increases on cigarette prices in South Africa. Tob Control 2018;27:65–71. 10.1136/tobaccocontrol-2016-053340 28341767 PMC5801652

[10] Laslett A-M, Jiang H, Room R. Minimum unit price deters heaviest alcohol purchasers. Lancet Public Health 2021;6:e535–6. 10.1016/S2468-2667(21)00095-5 34058126

[11] Anderson P, O'Donnell A, Kaner E, et al. Impact of minimum unit pricing on alcohol purchases in Scotland and Wales: controlled interrupted time series analyses. Lancet Public Health 2021;6:e557–65. 10.1016/S2468-2667(21)00052-9 34058125

[12] Western Cape Government. Western Cape alcohol-related harms reduction policy: White Paper. In: Department of the premier. Cape Town: Western Cape Government, 2017.

[13] Republic of South Africa. Budget review 2021. National Treasury, 2021.

[14] Gibbs N, Angus C, Dixon S, et al. Effects of minimum unit pricing for alcohol in South Africa across different drinker groups and wealth Quintiles: a modelling study. BMJ Open 2021;11:e052879. 10.1136/bmjopen-2021-052879 PMC8354280 34373316

[15] Van Walbeek C, Chelwa G. The case for minimum unit prices on alcohol in South Africa. S Afr Med J 2021;111:680–4. 10.7196/SAMJ.2021.v111i7.15430 34382553

[16] Statistics South Africa. Facts you might not know about social grants, 2016. Available: http://www.statssa.gov.za/?p=7756

[17] Nwosu CO, Oyenubi A. Income-Related health inequalities associated with the coronavirus pandemic in South Africa: a decomposition analysis. Int J Equity Health 2021;20:1–12. 10.1186/s12939-020-01361-7 33413442 PMC7790046

[18] Ataguba JE-O. Alcohol policy and taxation in South Africa. Appl Health Econ Health Policy 2012;10:65–76. 10.2165/11594860-000000000-00000 22136105

[19] Stiglitz JE, Rosengard JK. Economics of the public sector: fourth International student edition. WW Norton & Company, 2015.

[20] Summers LH. Taxes for health: evidence clears the air. Lancet 2018;391:1974–6. 10.1016/S0140-6736(18)30629-9 29627162

[21] Verguet S, Kearns PKA, Rees VW. Questioning the regressivity of tobacco taxes: a distributional accounting impact model of increased tobacco taxation. Tob Control 2021;30:245–57. 10.1136/tobaccocontrol-2019-055315 32576701 PMC8077213

[22] Saxena A, Stacey N, Puech PDR, et al. The distributional impact of taxing sugar-sweetened beverages: findings from an extended cost-effectiveness analysis in South Africa. BMJ Glob Health 2019;4:e001317. 10.1136/bmjgh-2018-001317 PMC6730580 31543983

[23] Verguet S, Gauvreau CL, Mishra S, et al. The consequences of tobacco tax on household health and finances in rich and poor smokers in China: an extended cost-effectiveness analysis. Lancet Glob Health 2015;3:e206–16. 10.1016/S2214-109X(15)70095-1 25772692

[24] Verguet S, Kim JJ, Jamison DT. Extended cost-effectiveness analysis for health policy assessment: a tutorial. Pharmacoeconomics 2016;34:913–23. 10.1007/s40273-016-0414-z 27374172 PMC4980400

[25] Briggs ADM, Wolstenholme J, Blakely T, et al. Choosing an epidemiological model structure for the economic evaluation of non-communicable disease public health interventions. Popul Health Metr 2016;14:17. 10.1186/s12963-016-0085-1 27152092 PMC4857239

[26] Republic of South Africa. Guidelines for pharmacoeconomic submissions. Department of Health, Government Gazette, 2012.

[27] International Monetary Fund. Countries at a glance: South Africa, 2021. Available: https://www.imf.org/en/Countries/ZAF#ataglance

[28] Watkins DA, Olson ZD, Verguet S, et al. Cardiovascular disease and impoverishment averted due to a salt reduction policy in South Africa: an extended cost-effectiveness analysis. Health Policy Plan 2016;31:75–82. 10.1093/heapol/czv023 25841771 PMC4724166

[29] Thørrisen MM, Bonsaksen T, Hashemi N, et al. Association between alcohol consumption and impaired work performance (presenteeism): a systematic review. BMJ Open 2019;9:e029184. 10.1136/bmjopen-2019-029184 PMC6661906 31315869

[30] Van Walbeek C, Blecher E. The economics of alcohol use, misuse and policy in South Africa South Africa: WHO South Africa Office, 2014. Available: http://www.tobaccoecon.uct.ac.za/sites/default/files/image_tool/images/405/People/the-economics-of-alcohol-policy-in-south-africa.pdf

[31] World Bank. DataBank: global economic monitor (GEM), 2021. Available: https://databank.worldbank.org/source/global-economic-monitor-(gem)

[32] Ataguba JE, McIntyre D. Paying for and receiving benefits from health services in South Africa: is the health system equitable? Health Policy Plan 2012;27 Suppl 1:i35–45. 10.1093/heapol/czs005 22388498

[33] Hatcher AM, Gibbs A, McBride R-S, et al. Gendered syndemic of intimate partner violence, alcohol misuse, and HIV risk among peri-urban, heterosexual men in South Africa. Soc Sci Med 2019;112637:112637. 10.1016/j.socscimed.2019.112637 PMC7296316 31708236

[34] Ramsoomar L, Gibbs A, Chirwa ED, et al. Pooled analysis of the association between alcohol use and violence against women: evidence from four violence prevention studies in Africa. BMJ Open 2021;11:e049282. 10.1136/bmjopen-2021-049282 PMC8314692 34312207

[35] Sassi F, Belloni A, Mirelman AJ, et al. Equity impacts of price policies to promote healthy behaviours. Lancet 2018;391:2059–70. 10.1016/S0140-6736(18)30531-2 29627166 PMC6642722

[36] Navsaria PH, Nicol AJ, Parry CDH, et al. The effect of lockdown on intentional and nonintentional injury during the COVID-19 pandemic in Cape town, South Africa: a preliminary report. S Afr Med J 2021;111:110–3. 10.7196/SAMJ.2021.v111i2.15318 33944719

[37] Holmes J, Meng Y, Meier PS, et al. Effects of minimum unit pricing for alcohol on different income and socioeconomic groups: a modelling study. The Lancet 2014;383:1655–64. 10.1016/S0140-6736(13)62417-4 PMC4018486 24522180

[38] Vandenberg B, Sharma A. Are alcohol taxation and pricing policies regressive? Product-level effects of a specific Tax and a minimum unit price for alcohol. Alcohol Alcohol 2016;51:493–502. 10.1093/alcalc/agv133 26719379

[39] Shield K, Manthey J, Rylett M, et al. National, regional, and global burdens of disease from 2000 to 2016 attributable to alcohol use: a comparative risk assessment study. Lancet Public Health 2020;5:e51–61. 10.1016/S2468-2667(19)30231-2 31910980

[40] Mutyambizi C, Pavlova M, Hongoro C, et al. Incidence, socio-economic inequalities and determinants of catastrophic health expenditure and impoverishment for diabetes care in South Africa: a study at two public hospitals in Tshwane. Int J Equity Health 2019;18:1–15. 10.1186/s12939-019-0977-3 31118033 PMC6530010

[41] Van Walbeek C, Chelwa G. Using price-based interventions to reduce abusive drinking in the Western Cape Province 2019.

[42] Matzopoulos RG, Prinsloo M, Butchart A, et al. Estimating the South African trauma caseload. Int J Inj Contr Saf Promot 2006;13:49–51. 10.1080/15660970500036382 16537225

[43] Maffessanti A, Lee-Angell E. HIV absenteeism study. South Africa: AIC Insurance Company and Welfitt Oddy, 2005.

[44] Bola S, Dash I, Naidoo M, et al. Interpersonal violence: quantifying the burden of injury in a South African trauma centre. Emerg Med J 2016;33:208–12. 10.1136/emermed-2014-204160 26362579

[45] Parkinson F, Kent SJW, Aldous C, et al. The hospital cost of road traffic accidents at a South African regional trauma centre: a micro-costing study. Injury 2014;45:342–5. 10.1016/j.injury.2013.04.007 23731494

[46] Matzopoulos RG, Truen S, Bowman B, et al. The cost of harmful alcohol use in South Africa. S Afr Med J 2014;104:127–32. 10.7196/samj.7644 24893544

[47] Tangka FK, Trogdon JG, Nwaise I, et al. State-level estimates of cancer-related absenteeism costs. J Occup Environ Med 2013;55:1015–20. 10.1097/JOM.0b013e3182a2a467 23969498 PMC4731096

