# Supplementary material for: Equity impact of minimum unit pricing of alcohol on household health and finances among rich and poor drinkers in South Africa
Source: BMJ Glob Health. 2022 Jan 6;7(1):e007824. doi: 10.1136/bmjgh-2021-007824 (PMC8739056; doi:10.1136/bmjgh-2021-007824)
Supplement: Supplementary data [file bmjgh-2021-007824supp001.pdf]

Supplementary Webappendix

of

Equity impact of minimum unit pricing of alcohol on household health and finances in rich and poor drinkers in South Africa

by

N.K. Gibbs, C. Angus, S.Dixon, C.D.H. Parry, P.S. Meier, M.K. Boachie, S. Verguet

In this supplementary webappendix, we report on the detailed inputs and assumptions that were used in the application of our minimum unit pricing (MUP) policy model, for which we heavily drew from the previously published analysis by Gibbs et al. (2021) (1).

1. Description of the data sources used for the comprehensive policy model

We detail in Figure A1 below all the data sources used for the comprehensive policy model, expanded from a previously published figure by Gibbs et al. (2021)(1).

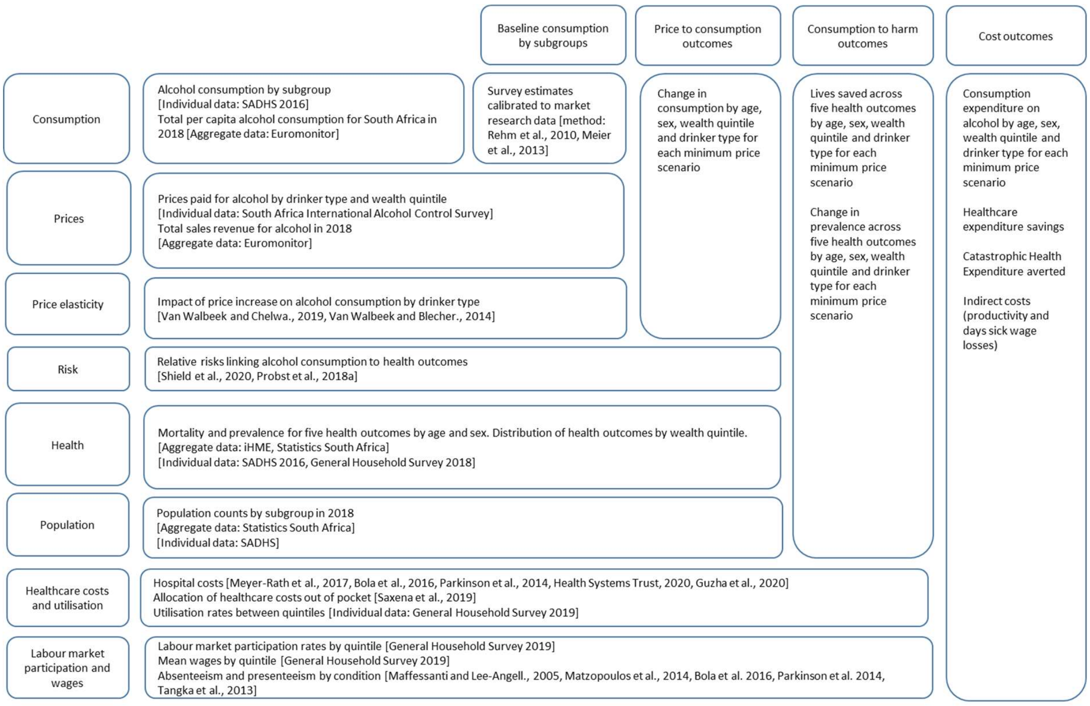

Figure A1. Detailed display of all the data sources used in the comprehensive policy model expanded in our study via extended cost-effectiveness analysis methods. Original source: Gibbs et al. (2021) (licensed under Creative Commons Attribution (CC BY 4.0)). (1)

2. Disease-related expenditures and data sources

We report in Table A1 below the inputs used for the estimation of disease- and injury-related expenditures, along with the corresponding data sources. All costs were adjusted to the year 2018.

Condition	Unit cost, per patient	Source
**HIV**	ZAR 3,319<br>(2017/18)	Meyer-Rath, van Rensburg (2). Conservative assumption of annual cost for first-line treatment.
**Intentional injury**	ZAR 58,928<br>(2013)	Bola, Dash (3).
**Road injury**	ZAR 56,592<br>(2012)	Parkinson, Kent (4).
**Liver cirrhosis**	R2,967<br>(2018)	Health Systems Trust (5). Conservative assumption of one patient day.
**Breast cancer**	Early stage: ZAR 14,915<br>Late stage: ZAR 16,869<br>(2015)	Guzha, Thebe (6).

Table A1. Inputs used for the estimation of disease- and injury-related expenditures, along with corresponding data sources. Note: for the unit cost per patient, the corresponding year is given in parentheses.

3. Adjusting the elasticities

The elasticities used in the original model were -0.40, -0.22 and -0.18 for moderate, occasional binge and heavy drinkers, respectively (7). We adjusted these elasticities to incorporate an income gradient using -0.86 and -0.50 elasticity for low and high socioeconomic status (SES) (8). To remain on the conservative side we considered the bottom two quintiles as low SES and the top three quintiles as high SES.

Drinker type	QI	QII	QIII	QIV	QV
Moderate	-0.53	-0.53	-0.31	-0.31	-0.31
Occasional binge	-0.29	-0.29	-0.17	-0.17	-0.17
Heavy drinkers	-0.24	-0.24	-0.14	-0.14	-0.14

Table A2. Price elasticities of demand for alcohol used in the comprehensive policy model.

4. Price shifting and elasticities

To simulate a minimum unit price (MUP) policy, each price distribution was changed so that any prices less than ZAR10 was moved up to exactly ZAR10, prices at or above ZAR10 per standard drink were left unchanged. This allowed the calculation of a new mean price and percentage change in mean price for each wealth/drinker group.

This conservative assumption assumes the industry response is to leave prices above the threshold unchanged: evidence of this was found in Scotland (9). However, if the price of products above the MUP level also increases, then the policy would be more effective, albeit somewhat less targeted.

The price change faced by different groups will depend on their purchases at baseline (before MUP policy). For example, groups who bought less of their alcohol below the threshold will experience less of a price increase.

Following the percentage change in price and using the appropriate elasticity enable the calculation of the new consumption levels in response to the change in prices created by the MUP policy. The price elasticity of demand can be written as follows:

$$\text{Price elasticity of demand}_{ij} = \frac{\frac{\text{new consumption}_{ij} - \text{baseline consumption}_{ij}}{\text{consumption}_{ij}}}{\frac{\text{new price}_{ij} - \text{baseline price}_{ij}}{\text{baseline price}_{ij}}}, \qquad \text{(A1)}$$

where i is drinker group and j is wealth quintile.

5. Health services utilisation rates

In this section, we detail the assumptions used for the healthcare utilisation rates for each of the five diseases and injuries examined in our study, by wealth quintile (QI=poorest; QV=richest).

HIV/AIDS

Using data from the General Household Survey (GHS) 2019 (10), we calculated quintile-specific utilisation rates by using the question on whether a respondent consulted a health worker as a result of illness in the last 30 days prior to the survey and HIV status. The overall figure (average) was 68% which compares well with the UN estimate of 70% of HIV patients on treatment (11).

QI	QII	QIII	QIV	QV
63.1%	71.4%	69.4%	60.5%	89.5%

Table A3. Healthcare utilisation rates used for HIV/AIDS across wealth quintiles.

Cancer/liver cirrhosis

The 2019 General Household Survey (10) provides data on those with cancer, but not breast or any specific cancer. Given that breast cancer ranks number one among all cancers in South Africa (12), we estimated that 0.3% would be the prevalence rate for breast cancer in 2019 based on the 2019 General Household Survey. Applying a similar approach used to obtain the HIV/AIDS utilisation rates (see immediately above), we estimated the number of breast cancer patients on treatment with the following quintile-specific estimates (Table A4).

QI	QII	QIII	QIV	QV
52.2%	55.7%	50.3%	67.7%	89.1%

Table A4. Healthcare utilisation rates used for cancer across wealth quintiles. Note: our original estimation with the 2019 General Household Survey[9] led to 100% for QI, which was unrealistic. Hence, we replaced this 100% value with the rate from "any condition" for QI.

As for liver cirrhosis, we used the utilisation rates corresponding to "any condition" (from the General Household Survey[9] questionnaire) as there were no other specific healthcare utilisation rate variables that could be identified (Table A5).

QI	QII	QIII	QIV	QV
**52.2%**	54.5%	53.5%	53.4%	63.2%

Table A5. Healthcare utilisation rates used for liver cirrhosis across wealth quintiles.

Intentional injury/road injury

The general healthcare utilisation rates (as calculated above in Table A5) were adjusted to account for how population prevalence of injury would translate to trauma admissions for either intentional or road injury. We used South African research documenting trauma admissions (from 1999; Matzopoulos et al. 2006[11]) combined with Global Burden of Disease (GBD) data (from the same year) (13) to derive a correspondence multiplier between prevalence and hospital admissions (Table A6).

Category in GBD	Prevalence (IHME 1999)	Category in Matzopoulos et al. (2006)	Number of cases	Estimated multiplier
**Transport injuries**	1,566,000	Traffic	302,900	0.19
**Unintentional injuries**	3,392,800	Other injuries	416,400	0.12
**Interpersonal violence and self-harm**	1,851,600	Violence	757,200	0.41

Table A6. Estimated correspondence multiplier between injury prevalence and admissions to hospital.

The estimated multipliers (Table A6) were then used to adjust the general healthcare utilisation rates (Table A5) in the following manner:

$$utilisation_{adj,qi} = \frac{utilisation_{qi}}{(\sum_{q=1}^{5} utilisation_{qi})/5} \times multiplier \,, \quad (A.2)$$

the results of which are reported in Table A7.

	QI	QII	QIII	QIV	QV
**Road injury**	0.18	0.19	0.18	0.18	0.22
**Intentional violence**	0.39	0.40	0.40	0.40	0.47

Table A7. Healthcare utilisation rates used for road injury and intentional violence, across wealth quintiles.

6. Absenteeism

In this section, we detail the assumptions made for the computation of absenteeism, that it the number of work days lost due to each of the five conditions examined in our study.

Gibbs N, *et al. BMJ Global Health* 2022; 7:e007824. doi: 10.1136/bmjgh-2021-007824

HIV/AIDS

A report by a South African insurance company states that those who have been diagnosed with HIV and who are being treated take 1,392 days (due to illness and treatment) out of 36,022 working days (14). Assuming a total of 252 working days in a year, this would equate to 14 work days lost per year.

Liver cirrhosis

Data taken from Matzopoulos et al. (2014) (15) stated that absenteeism rates averaged 2.3% in workers earning ZAR1,000 or less per month, and 1.3% in workers earning ZARR10,000 to 15,000 per month. The number of working days in South Africa per year is 252 days (16). We have therefore assumed 6 work days lost per year for the quintile I and 3 days lost per year for quintiles II, III, IV, and V.

Intentional injury and road injury

Here, the estimates of work days lost relate to the days spent in hospitalization due to these injuries. We drew corresponding estimates from microcosting studies on hospital costs (3, 4).

Breast cancer

Unfortunately, specific estimates for a South African setting (reviewing the published literature), or from a similar low- and middle-income country setting, could not be identified. Therefore, as a proxy, we extracted estimates from a US study corresponding to 6.1 work days lost per year (17).

Gibbs N, *et al. BMJ Global Health* 2022; 7:e007824. doi: 10.1136/bmjgh-2021-007824

References

1. Gibbs NK, Angus C, Dixon S, Parry CD, Meier PS. Effects of Minimum Unit Pricing for Alcohol in South Africa Across Different Drinker Groups and Wealth Quintiles: A Modelling Study. BMJ open. 2021;11(8).
2. Meyer-Rath G, van Rensburg C, Chiu C, Leuner R, Jamieson L, Cohen S. The per-patient costs of HIV services in South Africa: Systematic review and application in the South African HIV Investment Case. PloS one. 2019;14(2):e0210497.
3. Bola S, Dash I, Naidoo M, Aldous C. Interpersonal violence: quantifying the burden of injury in a South African trauma centre. Emergency medicine journal. 2016;33(3):208-12.
4. Parkinson F, Kent S, Aldous C, Oosthuizen G, Clarke D. The hospital cost of road traffic accidents at a South African regional trauma centre: A micro-costing study. Injury. 2014;45(1):342-5.
5. Health Systems Trust. District Health Barometer 2018/19. 2020.
6. Guzha N, Thebe T, Butler N, Valodia P. Development of a method to determine the cost of breast cancer treatment with chemotherapy at Groote Schuur Hospital, Cape Town, South Africa. South African Medical Journal. 2020;110(4):296-301.
7. van Walbeek C, Chelwa G. Using price-based interventions to reduce abusive drinking in the Western Cape Province. 2019.
8. Van Walbeek C, Blecher E. The economics of alcohol use, misuse and policy in South Africa South Africa: WHO South Africa Office; 2014 [Available from: http://www.tobaccoecon.uct.ac.za/sites/default/files/image_tool/images/405/People/the-economics-of-alcohol-policy-in-south-africa.pdf.
9. Stead. M, Critchlow. N, Eadie. D, Fitzgerald. N, Angus. K, Purves. R, et al. Evaluating the impact of alcohol minimum unit pricing in Scotland: Observational study of small retailers. Stirling; 2020.
10. Statistics South Africa. General Household Suvery 2019 2020 [Available from: http://www.statssa.gov.za/publications/P0318/P03182019.pdf.
11. UNAIDS. UNAIDS South Africa: Overview online2020 [Available from: https://www.unaids.org/en/regionscountries/countries/southafrica.
12. International Agency for Research on Cancer. The Global Cancer Observatory (GCO) 2020 [Available from: https://gco.iarc.fr/.
13. (IHME) IfHMaE. GBD Compare Seattle, WA: IHME, University of Washington; 2019 [Available from: http://vizhub.healthdata.org/gbd-compare.
14. Maffessanti A, Lee-Angell E. HIV Absenteeism Study. South Africa: AIC Insurance Company and Welfitt Oddy; 2005.
15. Matzopoulos RG, Truen S, Bowman B, Corrigall J. The cost of harmful alcohol use in South Africa. South African Medical Journal. 2014;104(2):127-32.
16. Excelnotes. Working days in South Africa 2021 2021 [Available from: https://excelnotes.com/working-days-south-africa-2021/#:~:text=In%20South%20Africa%2C%20there%20are,Day%20and%20Day%20of%20Goodwill.
17. Tangka FK, Trogdon JG, Nwaise I, Ekwueme DU, Guy Jr GP, Orenstein D. State-level estimates of cancer-related absenteeism costs. Journal of occupational and environmental medicine/American College of Occupational and Environmental Medicine. 2013;55(9):1015.
